# Histopathological Findings of Lung Cancer in Black Coal Miners in the Czech Republic

**DOI:** 10.1093/eurpub/ckac130.089

**Published:** 2022-10-25

**Authors:** H Tomaskova, J Horacek, H Slachtova, A Splichalova, P Riedlova, A Dalecka, Z Jirak, R Madar

**Affiliations:** 1 Department of Epidemiology and Public Health, Faculty of Medicine, University of Ostrava, Ostrava, Czechia; 2 Public Health Institute Ostrava, Ostrava, Czechia; 3 Centre for Epidemiological Research, Faculty of Medicine, University of Ostrava, Ostrava, Czechia; 4 Institute of Molecular and Clinical Pathology, Faculty of Medicine, University of Ostrava, Ostrava, Czechia

## Abstract

**Background:**

Coal miners with coal workers’ pneumoconiosis (CWP, J60 according to ICD-10) were previously found to have a significantly higher risk of lung carcinoma compared to the general male population. The presented study aimed to analyze the incidence of lung carcinoma in miners, histopathological findings in cohorts with and without CWP, and effect of smoking cessation on the histopathological profile.

**Methods:**

Analysed cohorts consisted of miners with (n = 3476) and without (n = 6687) CWP. Data on personal and working history obtained from the medical records were combined with information on lung cancer from the Czech Oncological Register and histopathological findings. Statistical analysis was performed using non-parametric tests and the incidence risk ratio (significance level of 5%).

**Results:**

In 1992-2015, 180 miners (2.7%) without CWP and 169 (4.9%) with CWP, respectively, were diagnosed with lung carcinoma. The risk of lung cancer in miners with CWP was 1.82 (95% CI: 1.48-2.25) times higher than in those without CWP. Squamous cell carcinoma (37%) was the most common histopathological type, followed by adenocarcinoma (22%) and small cell carcinoma (21%). A statistically significant difference between the cohorts (p = 0.003) was found in the histopathological subtypes, with the incidence of small cell carcinoma being 2 times higher in miners without CWP than in those with CWP. Only a few individuals with lung carcinoma were non-smokers. The incidence of small cell carcinoma, which is strongly associated with smoking, is significantly higher in miners without CWP.

**Conclusions:**

Smoking constitutes the most important risk factor for developing lung carcinoma even in that cohort. However, CWP remains a very important risk factor.

This work was supported by grant of Research Support Foundation, Vaduz. Markus R. Tödtli Consulting and project Healthy Aging in the Industrial Environment CZ.02.1.01/0.0/0.0/16_019/0000798 (HAIE).

**Key messages:**

- The risk of lung cancer in black coal miners with coal workers’ pneumoconiosis (CWP) was 1.82 (95% CI: 1.48–2.25) times higher than in those without CWP.
- The incidence of small cell carcinoma was 2 times higher in black coal miners without CWP than in those with CWP.

